# The impact of age on clinicopathological features and treatment results in patients with localised prostate cancer receiving definitive radiotherapy

**DOI:** 10.2340/1651-226X.2024.40759

**Published:** 2024-11-05

**Authors:** Cem Onal, Ozan Cem Guler, Aysenur Elmali, Birhan Demirhan, Melek Yavuz

**Affiliations:** a Department of Radiation Oncology, Adana Dr. Turgut Noyan Research and Treatment Center, Baskent University Faculty of Medicine, Adana, Turkey; b Department of Radiation Oncology, Baskent University Faculty of Medicine, Ankara, Turkey; c Division of Radiation Oncology, Iskenderun Gelisim Hospital, Hatay, Turkey

**Keywords:** Age, prostate cancer, radiotherapy, survival, toxicity

## Abstract

**Background:**

This study assessed the biochemical disease-free survival (bDFS), prostate cancer-specific survival (PCSS), overall survival (OS), and side effects in patients aged < 70 and ≥ 70 years following definitive radiotherapy (RT). It also analysed the correlation between age at diagnosis and clinicopathological characteristics of prostate cancer (PCa).

**Methods:**

The prognostic factors for bDFS, PCSS, and OS were determined through univariable and multivariable analyses. Two age groups were also compared in terms of acute and late grade ≥ 2 genitourinary (GU) and gastrointestinal (GI) toxicities, the predictors of which were determined through logistic regression analysis.

**Results:**

Of the 1,328 patients, 715 (53.8%) and 613 (46.2%) were aged < 70 and ≥ 70 years, respectively. Median follow-up time was 84.5 months. No significant differences in the 7-year bDFS (86.3% vs. 86.8%) and PCSS rates (92.9% vs. 93.3%) were found between the ≥ 70 and < 70 age groups. The multivariable analysis showed that advanced clinical T stage, high International Society of Urological Pathology (ISUP) grade, and high-risk disease independently predicted poor bDFS and PCSS. Metastatic lymph nodes were another bDFS prognostic factor. The multivariable analysis identified age ≥ 70 years, cardiac events at diagnosis, advanced stage, higher ISUP grade, and non-use of simultaneous integrated boost technique as negative factors for OS. Additionally, diabetes and transurethral resection of the prostate (TUR-P) independently predict late-grade ≥ 2 GU toxicity.

**Interpretation:**

Definitive RT is a safe and effective treatment for patients with localised PCa no matter their age. Although patients over 70 years have higher risk factors and comorbidities, their bDFS, PCSS, and toxicities were comparable to those of patients aged < 70 years.

## Background

Prostate cancer (PCa) is the second most prevalent cancer and the fifth most common cause of cancer-related deaths in men globally [1]. The median age at diagnosis is 66 years, but the highest PCa incidence occurs in men aged 70–74 years [2]. As the proportion of > 65-year-old in population increases, PCa prevalence is expected to rise, making it a growing public health concern.

Older age at diagnosis has been associated with poor prognosis for PCa in some observational studies, whereas other studies suggested that the effect of age depends on the treatment method [3–5]. In a recent review, Shaheen et al. [5] indicated that age ≥ 70 years could have a negative impact on the prognosis of patients undergoing radical prostatectomy (RP), but could have a positive impact on the prognosis of men receiving external beam radiotherapy (RT). A secondary analysis of randomised studies conducted by the Radiation Therapy Oncology Group (RTOG) showed that PCa patients aged ≥ 70 years had better outcomes in terms of distant metastasis (DM), biochemical control, and survival rates when treated with RT compared to patients aged < 70 years [6].

Although age is often regarded as a possible prognostic factor in numerous studies, elderly patients are frequently not well represented in clinical trials, resulting in a sparse evidence base [7, 8]. In observational studies, determining the relationship between diagnosis at older age and worse prognosis is challenging because most population-based studies do not report comprehensive data on clinicopathologic factors, comorbidity, and toxicity. Furthermore, patient age is a selection criterion in most clinical trials, with older age being an exclusion factor.

Thus, our objective was to assess any potential differences in clinicopathological characteristics and comorbidities, as well as treatment outcomes and toxicity results, among patients aged < 70 years and ≥ 70 years undergoing definitive RT. Based on the aforementioned findings, treatment outcomes including biochemical disease-free survival (bDFS), prostate cancer-specific survival (PCSS), overall survival (OS), and treatment-related toxicity were assessed in patients aged < 70 and ≥ 70 years.

## Materials and methods

### Patient selection

All patients were treated according to the institutional protocol and provided informed consent prior to treatment. Patients who were lost to follow-up were excluded from the study. We analysed the clinical data of 1,328 patients diagnosed with localised PCa treated with definitive RT between March 2010 and November 2021. All patients received treatment according to the institutional protocol [9]. The D’Amico Criteria were used to stratify the patients according to their pre-treatment serum prostate specific antigen (PSA) level, clinical stage, and biopsy-based Gleason score [10]. The International Society of Urological Pathology (ISUP) system was utilised for histological grading.

The inclusion criteria were as follows: confirmed histological diagnosis of prostate adenocarcinoma, curative-intent definitive RT, and an Eastern Cooperative Oncology Group performance score of 0–1. Patients were excluded if they had undergone RP, had DM, and had transurethral resection of the prostate (TUR-P) within 3 months before RT. Moreover, patients lost to follow-up were excluded from the study. Patients with a minimum of 18 months of follow-up were included in the study, with the most recent follow-up occurring in April 2023.

According to the American Joint Committee on Cancer 8th edition staging manual, metastasis to pelvic lymph nodes – including the pelvic, hypogastric, obturator, iliac, and sacral nodes – is classified as N1 disease. Only patients with or without clinical N1 disease were included.

This study was approved by the Baskent University Institutional Review Board (Project No. KA23/20) and supported by the Baskent University Research Fund. A written informed consent was obtained from all patients.

### Treatment planning

All patients received definitive RT through intensity-modulated radiation therapy (RT) or volumetric modulated arc therapy delivered in 39 fractions using a conventional fractionation scheme. In low-risk patients, the clinical target volume (CTV) included the prostate only. In intermediate- and high-risk patients, it involved the prostate and the proximal two-thirds of the seminal vesicles. The planning target volume (PTV) was generated by enlarging the CTV by a 5 mm margin towards the back and by an 8 mm margin in all other directions [9, 11]. Before 2012, the recommended radiation dose to be delivered to the prostate was 76–78 Gy. However, in 2012, the treatment approach was modified to incorporate a simultaneous integrated boost (SIB) to an intraprostatic lesion [9]. The high-risk patients received a pelvic nodal radiation at a dose ranging from 46 Gy to 54 Gy. The Monaco Treatment Planning System (Elekta Ltd., Crawley, UK) utilised the Monte Carlo algorithm to create plans, which were administered using a Versa-HD linear accelerator (Elekta AB, Stockholm, Sweden).

An genitourinary radiologist with over 25 years of experience delineated the intraprostatic lesions based on several criteria: digital rectal examination, a pathological biopsy with four quadrants and at least 12 core biopsies, and the presence of focal low-signal-intensity areas in the peripheral and/or transition zones. T2-weighted, diffusion-weighted imaging, and dynamic contrast-enhanced imaging sequences were utilised to contour all visible lesions.

Androgen deprivation therapy (ADT) was administered based on the treating physician’s assessment and patient preferences, and it was advised for patients with intermediate- and high-risk diseases. Patients with intermediate-risk disease were recommended to receive 6–12 months of ADT, which could involve luteinising hormone-releasing hormone agonists alone or in combination with an anti-androgen. For those with high-risk disease, 24–36 months of ADT was recommended.

### Follow-up

The primary endpoints were bDFS, which is the period from diagnosis to BF, and PCSS, which is the period from diagnosis to cancer-related death or to the most recent follow-up. The secondary outcomes were OS, which is the difference between the date of diagnosis and the date of death or last follow-up, and acute and late toxicities in the genitourinary (GU) and gastrointestinal (GI) systems.

The patients underwent regular monitoring during the study, with follow-up visits every 3 months for the first 2 years, every 6 months from years 3–5, and annually thereafter. According to the Phoenix criteria, biochemical failure (BF) was determined as PSA nadir plus 2 ng/mL [12]. Clinical progression was defined as the development of locoregional recurrence (LR) or DM based on imaging assessments. Toxicities were classified as either acute (within 90 days after starting RT) or late (more than 90 days after starting RT). The GI and GU toxicities were assessed based on the Common Terminology Criteria for Adverse Events version 4.0.2.

### Statistical analysis

Statistical analysis was performed using Statistical Package for the Social Sciences (SPSS) 26.0 (IBM Corp., Armonk, N.Y., USA) and GraphPad Prism version 10.1.2 (GraphPad Software, Inc., San Diego, CA). The patient and tumour characteristics of the < 70 and ≥ 70 age groups were analysed using χ^2^ test or Student’s *t*-test. The bDFS, PCSS, and OS rates were determined using the Kaplan–Meier method. PCa mortality is defined as death directly attributable to PCa, rather than death from other causes. However, if a patient with PCa progression or metastasis at the last visit dies from another cause, this case is also considered as PCa mortality. The log-rank test was utilised for univariable analysis. Covariates with a *p* < 0.05 in the univariable analysis were incorporated into the Cox proportional hazards model for the multivariable analysis. Univariable logistic regression was used to identify the predictors of late grade ≥ 2 GU and GI toxicities. The logistic regression analysis included the possible factors identified in the univariable analysis for the multivariable analysis. A two-sided *p* < 0.05 indicated statistical significance.

## Results

### Patient characteristics

**Figure 1:**
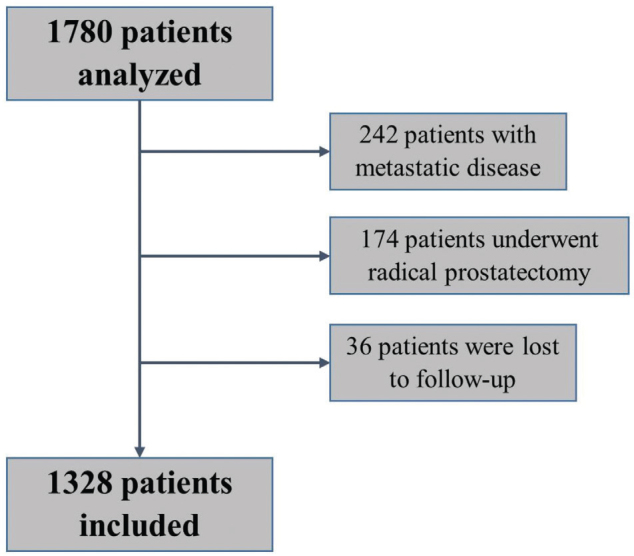
Flowchart for patient selection.

A total of 1,780 patients were treated during the study period. Of these, 242 were excluded because of DM, 174 had undergone RP, and 36 were lost to follow-up. Consequently, 1,328 patients were included in the study analysis (Figure 1). Table 1 summarises the patient and tumour characteristics of the entire patient population and according to age group. Of the 1,328 patients, 715 (53.8%) and 613 (46.2%) were aged < 70 and ≥ 70 years, respectively. The median age for the entire cohort was 69 years (range, 45–85 years), 64 years (range, 45–69 years) for the < 70 age group, and 74 years (range, 70–89 years) for the ≥ 70 age group. Those in the ≥ 70 age group exhibited significantly elevated rates of comorbidities, including cardiac disease and diabetes. Moreover, the ≥ 70 age group showed greater prevalence of high ISUP grades and higher incidence of high-risk diseases than the < 70 age group, and the difference was significant. Additionally, the number of patients who had undergone TUR-P and had received ADT was higher in the ≥ 70 age group than in the < 70 age group.

**Table 1:** Patient and tumour characteristics of the entire patient population and according to age groups.

Characteristics	Entire group *n* (%)	< 70 years *n* (%)	≥ 70 years *n* (%)	*p*
**Initial PSA (ng/mL, median, range)**	13.1 (1.1–1032.5)	12.2 (1.1–1032.5)	14.1 (2.3–912.6)	0.73
**Cardiac disease**				
Yes	656 (49.4)	285 (39.9)	371 (60.5)	< 0.001
No	672 (50.6)	430 (60.1)	242 (39.5)	
**Diabetes mellitus**				
Yes	222 (16.7)	103 (14.4)	119 (19.4)	0.02
No	1106 (83.3)	612 (85.6)	494 (80.6)	
**No of comorbidities**				
0	503 (37.9)	340 (47.6)	163 (26.6)	< 0.001
1	545 (41.0)	261 (36.5)	284 (46.3)	
2	217 (16.3)	91 (12.7)	126 (20.6)	
3	63 (4.7)	23 (3.2)	40 (6.5)	
**T stage**				
T1c	128 (9.6)	73 (10.2)	55 (9.0)	0.19
T2a	245 (18.4)	153 (21.4)	92 (15.0)	
T2b	299 (22.5)	145 (20.3)	154 (25.1)	
T2c	278 (20.9)	133 (18.6)	145 (23.7)	
T3a	152 (11.5)	85 (11.9)	67 (10.9)	
T3b	226 (17.1)	126 (17.6)	100 (16.3)	
**N stage**				
N0	1149 (86.5)	609 (85.2)	540 (88.1)	0.13
N1	179 (13.5)	106 (14.8)	73 (11.9)	
**ISUP grade**				
1	519 (39.1)	310(43.4)	209 (34.1)	0.003
2	271 (20.4)	142 (19.9)	129 (21.0)	
3	246 (18.5)	113 (15.8)	133 (21.7)	
4	138 (10.4)	65 (9.1)	73 (11.9)	
5	154 (11.6)	85 (11.9)	69 (11.3)	
**TUR**				
Yes	217 (16.3)	74 (10.3)	143 (23.3)	< 0.001
No	1111 (83.7)	641 (89.7)	470 (76.7)	
**Risk group**				
Low	340 (25.6)	216 (30.2)	124 (20.2)	< 0.001
Intermediate	403 (30.3)	202 (28.3)	201 (32.8)	
High	585 (44.1)	297 (41.5)	288 (47.0)	
**Irradiation field**				
Prostate only	775 (58.4)	426 (59.6)	349 (57.0)	0.67
Prostate + pelvic field	553 (41.6)	289 (40.4)	264 (43.0)	
**SIB**				
Yes	734 (55.3)	407 (56.9)	327 (53.3)	0.2
No	594 (44.7)	308 (43.1)	286 (46.7)	
**ADT**				
Yes	876 (66.0)	439 (61.4)	437 (71.3)	< 0.001
No	452 (34.0)	276 (38.6)	176 (28.7)	

PSA: prostate specific antigen; ISUP: International Society of Urological Pathology; TUR: transurethral resection; SIB: simultaneous integrated boost; ADT: androgen deprivation therapy.

For distant disease staging, conventional imaging modalities, including bone scintigraphy and computed tomography (CT) or magnetic resonance imaging (MRI), were used in 938 patients (71.6%), while 390 patients (29.4%) underwent prostate-specific membrane antigen–positron emission tomography/CT (PSMA-PET/CT).

The high-risk PCa patients were recommended to undergo pelvic nodal irradiation; however, 38 patients (6.5% of all high-risk patients) underwent prostate and seminal vesicle irradiation instead. Out of these 38 patients who underwent prostate-only irradiation, 29 (76.3%) were ≥ 70 years old and the remaining 9 (23.7%) were < 70 years old (*p* = 0.01). The 6 other PCa patients (1.0%) who did not receive ADT were ≥ 70 years old, whereas all high-risk PCa patients aged < 70 years received ADT.

### Patient outcomes

The median follow-up time for the entire cohort was 84.5 months [interquartile range (IQR) = 80.0–90.0 months]. The 7-year bDFS, PCSS, and OS rates were 86.5%, 93.1%, and 76.9%, respectively.

**Figure 2:**
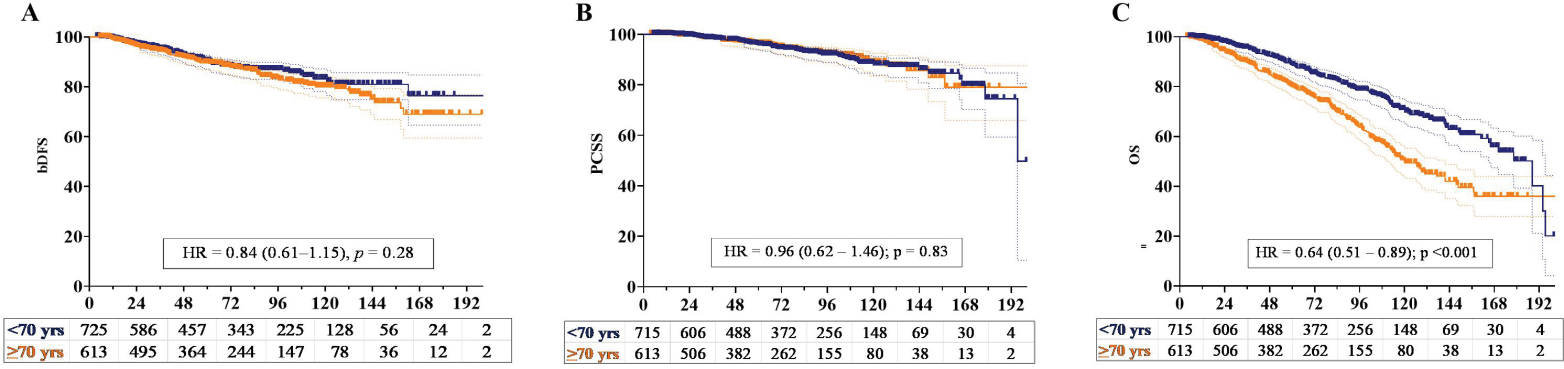
Kaplan–Meier plots of the (a) biochemical disease-free survival (bDFS), (b) prostate cancer-specific survival (PCSS), and (c) overall survival (OS) of patients aged < 70 (blue line) and ≥ 70 (yellow line) years.

Disease progression, occurring at a median of 35.6 months (1.1–159.6 months) after completion of RT, was observed in 177 patients (13.3%). Among these patients, 115 (8.7%) had DM only, 13 (1.0%) had LR only, and 21 (1.6%) had both DM and LR. Of the total, only 28 patients (2.1%) exhibited BF only with no signs of DM or LR. The 5-year LR rates were 2.3% and 1.6% for patients aged ≥ 70 and < 70 years, respectively, and this difference was not statistically significant (*p* = 0.23) (Figure 2A). Similarly, no significant difference in the 5-year DM rates was noted between for patients aged ≥ 70 and < 70 years (10.1% vs. 8.7%; *p* = 0.52) (Figure 2B).

In patients with high-risk disease, no significant differences were observed in bDFS (hazard ratio [HR] = 0.62 [95% confidence interval [CI]; 0.23–1.69]; *p* = 0.35], PCSS (HR = 1.01 [95% CI; 0.37–2.77]; *p* = 0.98), and OS (HR = 0.61 [95% CI; 0.36–1.39]; *p* = 0.15) between those who underwent pelvic nodal irradiation and those who did not.

At the latest follow-up, 986 patients were alive [75 patients (5.6%) with disease] and 342 had died [88 patients (6.7%) with disease and 254 patients (19.4%) without disease]. The primary causes of death aside from PCa included cardiac events (129 patients, 9.7%), secondary tumours (54 patients, 4.1%), and chronic renal disease (24 patients, 1.8%). The 5-year non-PCa mortality rate was significantly higher in the ≥ 70 age group than in the < 70 age group (17.1% vs. 7.4%; *p* < 0.001) (Figure 2C). A significant difference in the occurrence of deaths from cardiac events was noted between the ≥ 70 and < 70 age groups (6.5% vs. 3.2%; *p* = 0.02). Of the 129 patients who died due to a cardiac event, 96 (74.4%) had received ADT and 33 (35.6%) had not (*p* < 0.001). No significant difference in secondary malignancy-related deaths was observed between the ≥ 70 and < 70 age groups (2.4% vs. 1.7%; *p* = 0.25).

### Prognostic factors

**Figure 3:**
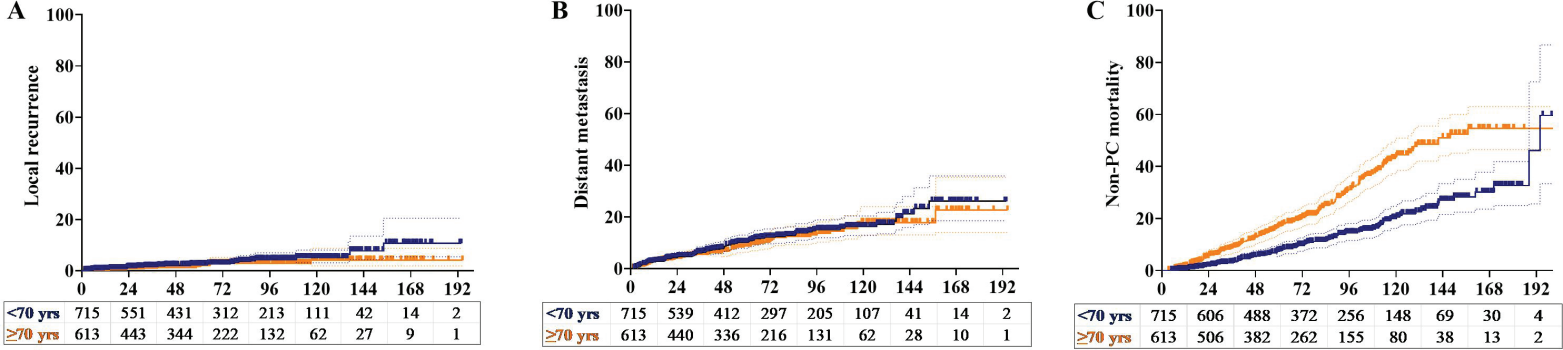
Kaplan–Meier plots of the (a) local recurrence, (b) distant metastasis, and (c) non-prostate cancer mortality in patients aged < 70 (blue line) and ≥ 70 (yellow line) years.

Serum PSA level, clinical T and N stages, ISUP grade, and risk group were the significant prognostic factors for bDFS and PCSS in the univariable analysis (Table 2). Another significant factor for PCSS was the utilisation of the SIB technique. No significant differences in the 7-year bDFS (86.3% vs. 86.8%; *p* = 0.28) and PCSS rates (92.9% vs. 93.3%; *p* = 0.83) were found between the ≥ 70 and < 70 age groups (Figure 3A–B). In the multivariable analysis, advanced clinical T stage, high ISUP grade, and high-risk disease were identified as independent predictors for poor bDFS and PCSS. Lymph node metastasis was also a significant prognostic factor for bDFS.

**Table 2:** Univariable and multivariable analyses of the prognostic factors for the biochemical disease-free survival (bDFS) and prostate cancer-specific survival (PCSS) of the entire cohort.

Patient characteristics	bDFS				PCSS			
	Univariate analysis		Multivariate analysis		Univariate analysis		Multivariate analysis	
	HR (95% CI)	*p*	HR (95% CI)	*p*	HR (95% CI)	*p*	HR (95% CI)	*p*
**Age**		0.28				0.83		
< 70 years	1				1			
≥ 70 years	0.84 (0.61–1.15)				0.96 (0.62–1.46)			
**Cardiac disease**		0.08				0.17		
Present	1				1			
Absent	1.32 (0.97–1.82)				1.35 (0.88–2.07)			
**Diabetes mellitus**		0.5				0.92		
Present	1				1			
Absent	0.87 (0.58–1.31)				0.92 (0.52–1.64)	-		
**PSA**		< 0.001		0.001		< 0.001		0.12
< 20 ng/mL	1		1	-	1		1	
≥ 20 ng/mL	3.67 (2.66–5.05)		2.07 (1.37–3.13)	-	4.52 (2.87–7.11)		1.47 (0.90–2.42)	
**ISUP grade**		< 0.001		< 0.001		< 0.001		< 0.001
1–3	1		1		1		1	
4–5	4.71 (3.44–6.43)		2.33 (1.58–3.44)		11.76 (7.43–18.61)		2.70 (1.61–4.54)	
**T stage**		< 0.001		0.001		< 0.001		< 0.001
< T3a	1		1		1		1	
≥ T3a	4.45 (3.25–6.10)		1.98 (1.31–3.00)		10.89 (6.59–17.99)		3.14 (1.73–5.70)	
**N stage**		< 0.001		< 0.001		< 0.001		0.17
N0	1		1		1		1	
N1	6.96 (5.06–9.58)		3.08 (2.10–4.53)		6.84 (4.44–10.53)		1.38 (0.87–2.19)	
**Risk group**								
Low	1		1		1		1	
Intermediate	2.41 (1.25–4.63)	0.008	1.12 (0.52–2.41)	0.78	1.79 (0.43–7.50)	0.42		
High	6.95 (3.92–12.33)	< 0.001	2.13 (1.10–4.12)	0.02	9.73 (6.24–12.65)	< 0.001	3.44 (2.08–5.68)	< 0.001
**SIB**		0.13				< 0.001		
Present	1				1			
Absent	1.28 (0.93–1.76)				2.77 (1.66–4.61)			

bDFS: biochemical disease-free survival; PCSS: prostate cancer specific survival; HR: hazard ratio; CI: confidence interval; PSA: prostate specific antigen; SIB: simultaneous integrated boost.

Patient age, cardiac disease status, serum PSA level, clinical T and N stages, ISUP grade, risk group, and utilisation of the SIB technique were the significant prognostic factors for OS in the univariable analysis (Supplementary Table). The 7-year OS rate was significantly higher in patients aged < 70 years than in their counterparts (82.5% vs. 70.0%; *p* < 0.001) (Figure 3C). In the multivariable analysis, age ≥ 70 years, any cardiac event at diagnosis, advanced stage, higher ISUP grade, and non-utilisation of the SIB technique were the negative factors for OS.

### Toxicity

**Figure 4:**
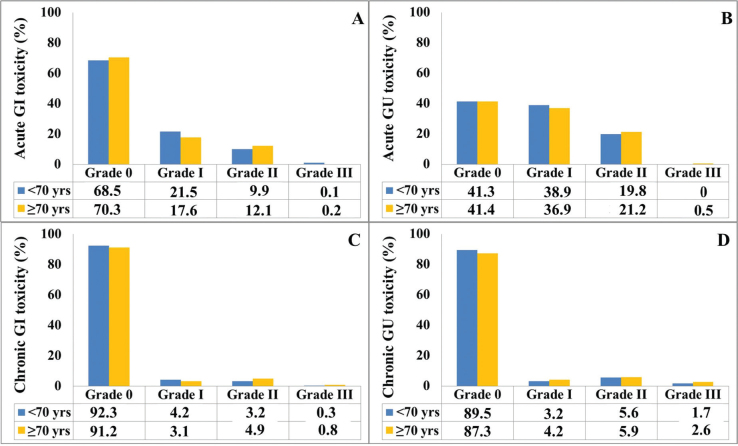
Bar graphs showing the acute (a) gastrointestinal (GI) and (b) genitourinary (GU) toxicities and the late (c) GI and (d) GU toxicities in patients aged < 70 (blue bar) and ≥ 70 (yellow bar) years.

No severe adverse events necessitated discontinuation of RT in any of the patients. Acute grade ≥ 2 GI toxicities occurred in 10.0% and 12.3% of the patients aged < 70 and ≥ 70 years, respectively, and the difference was not significant (*p* = 0.25) (Figure 4A). Only one patient experienced acute Grade 3 gastrointestinal (GI) toxicity, which involved rectal haemorrhage that required argon coagulation. In contrast, 145 patients had acute Grade 2 GI toxicity, primarily presenting as proctitis and diarrhoea. Additionally, no significant difference was observed in the incidence of acute Grade ≥ 2 genitourinary (GU) toxicities between patients aged ≥ 70 and those aged < 70 (19.8% vs. 21.7%; *p* = 0.42) (Figure 4B). Acute Grade 2 toxicities included cystitis in 201 patients, urgency in 60 patients, haematuria in six patients, and urinary incontinence in five patients. Additionally, three patients experienced acute Grade 3 haematuria. However, a borderline significant difference in late grade ≥ 2 GI toxicity was observed between the < 70 and ≥ 70 age groups (3.5% vs. 5.7%; *p* = 0.06), whereas no significant difference in late grade ≥ 2 GU toxicity was noted between the two groups (7.3% vs. 8.5%; *p* = 0.42) (Figure 3C–D). Acute Grade 2 toxicities included cystitis in 201 patients, urgency in 60 patients, haematuria in six patients, and urinary incontinence in five patients. Additionally, three patients experienced acute Grade 3 haematuria. No significant difference in the rates of urinary incontinence was observed between the age groups.

Clinical T stage, RT field, and ADT were independently correlated with late grade ≥ 2 GI toxicity in the univariable logistic regression analysis. Age and cardiac status showed a nearly significant impact on GI toxicity (Table 3). In the multivariable analysis, no significant predictor of late grade ≥ 2 GI toxicity was found, except for the presence of a cardiac event, which increases the late grade ≥ 2 GI toxicity to a nearly significant level.

**Table 3:** Univariable and multivariable logistic regression analysis of late grade ≥ 2 gastrointestinal (GI) and genitourinary (GU) toxicities.

Patient characteristics	Late ≥ 2 GI toxicity				Late ≥ 2 GU toxicity			
	Univariate analysis		Multivariate analysis		Univariate analysis		Multivariate analysis	
	OR (95% CI)	*p*	OR (95% CI)	*p*	OR (95% CI)	*p*	OR (95% CI)	*p*
**Age**		0.05		0.16		0.41		
< 70 years	1		1		1			
≥ 70 years	1.67 (0.99–2.83)		1.48 (0.86–2.53)		1.18 (0.79–1.76)			
**Cardiac disease**		0.05		0.07		0.46		
Present	1		1		1			
Absent	0.59 (0.35–1.01)		0.61 (0.35–1.05)		0.86 (0.58–1.28)			
**Diabetes mellitus**		0.31				0.01		0.008
Present	1				1		1	
Absent	0.71 (0.38–1.34)				0.54 (0.34–0.86)		0.52 (0.32–0.84)	
**TUR-P**		0.94				< 0.001		< 0.001
Present	1				1		1	
Absent	0.98 (0.49–1.95)				0.19 (0.12–0.28)		0.20 (0.13–0.30)	
**T stage**		0.003		0.62		0.02		0.88
< T3a	1		1		1		1	
≥ T3a	1.85 (1.09–3.14)		0.85 (0.46–1.60)		1.64 (1.08–2.48)		0.96 (0.57–1.62)	
**N stage**		0.4				0.09		
N0	1				1			
N1	0.70 (0.30–1.66)				1.59 (0.95–2.67)			
**RT field**		0.001		0.13		0.002		0.1
Prostate	1		1		1		1	
Prostate + pelvic field	2.39 (1.40–4.07)		1.74 (0.85–3.55)		1.90 (1.27–2.84)		1.65 (0.90–3.01)	
**SIB**		0.4				0.13		
Present	1				1			
Absent	1.25 (0.74–2.10)				1.37 (0.92–2.04)			
**ADT**		0.002		0.2		0.03		0.95
Present	1		1		1		1	
Absent	0.37 (0.19–0.73)		0.58 (0.25–1.33)		0.62 (0.39–0.97)		0.98 (0.54–1.80)	

OR: odds ratio; CI: confidence interval; TUR-P: transurethral resection of prostate; RT: radiotherapy; SIB: simultaneous integrated boost; ADT: androgen deprivation therapy.

Diabetes, TUR-P, clinical T stage, RT field, and ADT were independently correlated with late grade ≥ 2 GU toxicity in the univariable logistic regression analysis. In the multivariable logistic regression analysis, diabetes and TUR-P were identified as independent predictors of increased late grade ≥ 2 GU toxicity.

## Discussion

In this study, we found that ISUP grade and the prevalence of high-risk disease are higher in the ≥ 70 age group than in the < 70 age group; however, these two groups did not significantly differ in terms of bDFS or PCSS. This finding implies that advanced age should not preclude appropriate treatment, especially for those who have a reasonable quality of life, life expectancy, and performance status. Patients aged ≥ 70 years had a lower OS rate than their counterparts, even though both groups received nearly identical treatment modalities. Moreover, cardiac event-related deaths are more common in patients aged ≥ 70 years than in those aged < 70 years, emphasising the importance of non-PCa causes of deaths in this patient population. Furthermore, diabetes and TUR-P were independent factors that predict higher late grade ≥ 2 GU toxicity. Although incidences of diabetes and TUR-P were higher in patients aged ≥ 70 years than in those aged < 70 years, this patient population tolerated all treatments well, and no significant differences in toxicity rates were found between the two age groups. However, no significant predictor of late grade ≥ 2 GI toxicity was identified.

Age is a limiting factor in making treatment decisions for PCa patients. In most series, patients undergoing RT were older than those treated with RP, which may cause bias in treatment outcomes. Ideally, cancer characteristics, life expectancy determined by chronological age, comorbidity, and patient preferences must be considered in treatment selection. However, multiple studies have documented inadequate treatment of elderly PCa patients [3, 13]. Furthermore, significant variations in initial clinicopathological features, treatment approaches, and pre-treatment evaluations may exist based on age at diagnosis [4, 5].

Studies have reported different results regarding the prognostic significance of age in PCa patients undergoing different treatments. Bechis et al. [3] found a correlation between older age at diagnosis and worse prognosis for localised PCa in 11,790 men who underwent treatments such as RP, RT, watchful waiting, or ADT in the CaPSURE study. Similarly, an analysis of 160,787 men who underwent RP in a database study revealed a correlation between older age at diagnosis and increased PCa mortality rates [14]. In a nation-wide cohort study involving 121,392 PCa patients treated with RP, RT, deferred treatment, or ADT, Petterson et al. [4] found a strong inherent effect of age on the risk of PCa-related death. They found that advanced age was associated with an increased likelihood of PCa mortality in men undergoing various treatments. Interestingly, this trend was not observed in patients receiving RT, despite their having more severe tumour characteristics than those undergoing RP. In a recent review, Shaheen et al. [5] discovered that age impacts patient prognosis differently depending on the treatment method: it is a negative factor for patients undergoing RP and a positive factor for those treated with RT.

Some concerns have been raised regarding the true effect of age on treatment outcomes and survival in PCa patients treated with definitive RT, the majority of whom are elderly patients. Population-based studies have shown a decline in the proportion of patients receiving curative treatment by around age 65 [4]. In an institutional series, treatment decisions are typically based on patient performance status and life expectancy, similar to our study [15, 16]. Although studies have demonstrated that older patients undergoing RT had worse clinicopathological characteristics, the pathological results may be biased, as ‘true’ pathological findings are available only for RP-treated patients. In the present study, ISUP grade and the prevalence of high-risk disease are higher in the ≥ 70 age group than in the < 70 age group. However, these age groups showed no significant differences in terms of LR, DM, bDFS, and PCSS. One possible explanation is the increased utilisation of ADT in the ≥ 70 age group, and this could have potentially influenced the results. Older men might experience delayed testosterone recovery after ADT, and this factor alone could potentially lead to improved biochemical outcomes. Another potential explanation is that nearly all patients in the ≥ 70 age group received a planned treatment, with only 4.9% of the high-risk patients missing pelvic irradiation and only 1.0% lacking ADT. This explanation contradicts the fact that men who receive a curative-intent treatment began to decline in receiving curative treatment more sharply at around age 65, particularly those patients with intermediate- or high-risk PCa [4].

One significant issue for individuals over 70 is the presence of multiple health conditions that could lead to non-PCa mortality. Studies have shown that RT is associated with lower OS rates compared with RP. This difference is attributed to higher incidences of non-PCa causes of death, particularly cardiovascular events, in the RT group than in the RP group [17, 18]. In the present study, nearly 50% and 75% of the patients aged < 70 and ≥ 70 years, respectively, had comorbidities, most commonly cardiac events, diabetes, and chronic renal disease. In addition to older age, the presence of cardiac events, along with advanced T stage, high ISUP grade, and lack of SIB, had a negative impact on OS.

Population-based studies showed that ADT can elevate the risk of cardiovascular issues, especially in men with existing heart conditions [19, 20]. Contrarily, another research indicated that ADT does not elevate the likelihood of cardiac-related death in patients with clinically localised PCa [21]. In the present study, although the use of ADT did not have a significant impact on OS, 96 (74.4%) out of the 129 patients who died due to cardiac events had also been treated with ADT. This finding highlights the need for caution when using ADT in patients with cardiac disease at the time of diagnosis. However, this study lacks a robust risk group stratification for cardiovascular events and has limited ability to establish cause-and-effect relationships, including defining a causal link between ADT and cardiac mortality. Furthermore, secondary cancers may have an effect on other major causes of death, but no significant difference was observed between the investigated between patients aged ≥ 70 years and those aged < 70 years.

Toxicity outcomes in patients receiving definitive RT have not yet been thoroughly researched according to age groups. An earlier study involving a diverse group of patients older than 70 years found no increase in severe grade 3 to 4 side effects in more vulnerable or fragile patients [8]. In a subgroup analysis of the CHHiP trial, Wilson et al. [15] showed that hypofractionated RT was well tolerated by men aged ≥ 75 years. Our study did not find any significant difference in acute and late grade ≥ 2 GI and GU toxicities between the investigated age groups. Meanwhile, diabetes and TUR-P were associated with a higher risk of late grade ≥ 2 GU toxicity. Health-related quality of life (HRQoL) is a crucial outcome for older patients. However, the recruitment of individuals aged ≥ 75 years in most HRQoL trials has been limited, resulting in sparse data [22, 23]. An ongoing randomised trial, SPCG 19/GRand-P, which compares immediate curative therapy with conservative treatment in men aged ≥ 75 years with non-metastatic high-risk prostate cancer, will provide detailed information for this very elderly age group [24].

This study possesses some limitations. We analysed only those patients who completed their planned treatment and excluded those lost to follow-up. Additionally, we examined only those patients receiving definitive RT and not those undergoing RP or ADT. Additionally, regular cardiological or endocrinological monitoring was not performed to evaluate the harmful impacts of ADT on cardiovascular events and diabetes. Thus, additional prospective trials are necessary to more accurately evaluate the negative effects of ADT. Our study is a single-institution study that needs validation for a more accurate evaluation of clinical outcomes. Finally, we conducted an analysis comparing patients aged above and below 70 years. Further research incorporating additional age groups will be the focus of subsequent studies. However, despite its limitations, this study has advantages, including a large sample size, a relatively homogeneous patient population, use of modern RT techniques, prospective data collection with detailed comorbidities at diagnosis, and analysis of toxicity outcomes.

## Conclusion

Our analysis showed that definitive RT is a safe and effective treatment for patients with localized PCa irrespective of their age. Although patients aged ≥ 70 years are more likely to have high-risk factors and comorbidities than those aged < 70 years, no significant differences in bDFS, PCSS, and acute and late GI and GU toxicities were observed between these age groups. The common co-morbidities in patients undergoing definitive RT include age ≥ 70 years and presence of cardiovascular disease, which were identified as independent predictors of lower OS. This single-institution study necessitates validation to ensure that the findings have a significant clinical impact. Moreover, additional research is needed to substantiate the impact of age on clinicopathological characteristics, OS, and adverse effects, as well as the consequences of ADT and RT on non-PCa mortality.

## Supplementary Material

The impact of age on clinicopathological features and treatment results in patients with localised prostate cancer receiving definitive radiotherapy

## Data Availability

The data that support the findings of this study can be obtained from the corresponding author upon request.
