# Supplementary material for: The impact of age on clinicopathological features and treatment results in patients with localised prostate cancer receiving definitive radiotherapy
Source: Acta Oncol. 2024 Nov 5;63:40759. doi: 10.2340/1651-226X.2024.40759 (PMC11558863; doi:10.2340/1651-226X.2024.40759)
Supplement: The impact of age on clinicopathological features and treatment results in patients with localised prostate cancer receiving definitive radiotherapy [file AO-63-40759-s1.pdf]

Supplementary material has been published as submitted. It has not been copyedited, or typeset by Acta Oncologica

Supplementary Table. Univariate and multivariate analyses of prognostic factors for overall survival.

	**Overall survival**			
**Characteristics**	**Univariate analysis**		**Multivariate analysis**	
	**HR (95% CI)**	***p***	**HR (95% CI)**	***P***
**Patient characteristics**				
**Age**		<0.001		<0.001
**<70 years**	1		1	
**≥70 years**	1.85 (1.50–2.30)		1.78 (1.43–2.22)	
**Cardiac disease**		0.005		0.04
**Present**	1		1	
**Absent**	0.74 (0.60–0.91)		0.80 (0.64–0.99)	
**Diabetes mellitus**		0.12		
**Present**	1			
**Absent**	0.80 (0.61–1.06)			
**PSA**		<0.001		
**<20 ng/mL**	1		1	0.1
**≥20 ng/mL**	1.63 (1.32–2.02)		1.28 (0.96–1.72)	
**ISUP grade**		<0.001		0.002
**1–3**	1		1	
**4–5**	1.98 (1.56–2.51)		1.60 (1.19–2.15)	
**T stage**		<0.001		0.01
**<T3a**	1		1	
**≥T3a**	1.75 (1.40–2.18)		1.46 (1.09–1.95)	
**N stage**		<0.001		0.85
**N0**	1		1	
**N1**	1.53 (1.14–2.07)		1.03 (0.72–1.41)	
**Risk group**				
**Low**	1		1	
**Intermediate**	1.47 (1.07–2.03)	0.02	1.06 (0.69–1.65)	0.78
**High**	2.17 (1.63–2.89)	<0.001	1.30 (0.94–1.81)	0.11
**SIB**		<0.001		0.006
**Present**	1		1	
**Absent**	1.56 (1.24–1.96)		1.39 (1.10–1.76)	

Abbreviations: HR = hazard ratio, PSA = prostate specific antigen, SIB = simultaneous integrated boost, ADT = androgen deprivation therapy, RT = radiotherapy.
